# β-cell heterogeneity and molecular plasticity in type 2 diabetes: multi-omics perspectives and the role of gut microbiota

**DOI:** 10.3389/fcell.2025.1698296

**Published:** 2026-01-08

**Authors:** Evgeny Ruchko, Maria Chernysheva, Vasily Sokolov, Zakhar Starinnov, Marat Sabirov, Andrey Vasiliev

**Affiliations:** Cell Biology Laboratory, Koltzov Institute of Developmental Biology, Moscow, Russia

**Keywords:** GLP-1, gut microbiota, microbiome, pancreatic β-cells, probiotics, single-cell transcriptomics, type 2 diabetes

## Abstract

Type 2 diabetes (T2D) is a complex metabolic disorder characterized by systemic insulin resistance and progressive deterioration of pancreatic β-cell function. Advances in single-cell transcriptomics, epigenomics, and spatial transcriptomics have delineated marked β-cell heterogeneity, revealing subpopulations with differential secretory capacity, stress resilience, and vulnerability to metabolic and immune-mediated insults. These high-resolution approaches have further identified disease-associated alterations in other islet endocrine cells, as well as in immune, stromal, and exocrine pancreatic compartments, highlighting the central role of intercellular signaling in T2D pathogenesis. Concurrently, microbiome research has elucidated mechanisms by which gut microbial composition and metabolic activity modulate glucose homeostasis and β-cell function through immunoregulatory pathways, maintenance of epithelial barrier integrity, and enteroendocrine signaling, notably *via* glucagon-like peptide-1 (GLP-1). Therapeutic strategies targeting the gut microbiota include conventional probiotics, prebiotics, and fecal microbiota transplantation, alongside emerging synthetic biology approaches employing genetically engineered probiotic strains to deliver bioactive molecules, including GLP-1, directly in the gut microenvironment. This review integrates current multi-omics and experimental evidence to provide a comprehensive framework for understanding β-cell molecular plasticity, microbiota-mediated metabolic regulation, and their intersection as potential therapeutic targets. Such integrative approaches offer prospects for the development of precision interventions aimed at preserving or restoring β-cell function in T2D.

## 1 Introduction

T2D is a complex and progressive metabolic disorder driven not only by insulin resistance but also by the gradual loss of pancreatic β-cell functionality (Goyal et al., 2025). Declining insulin secretion and impaired adaptive capacity of β-cells are considered central elements in the pathogenesis of T2D, shaping both the clinical course and the success of therapeutic interventions. Despite available treatment options (Chong et al., 2024), there is no universal or sustainable approach to restoring β-cell function (Khunti et al., 2025), particularly after withdrawal of therapy, including metformin, insulin, and GLP-1 (Shoung et al., 2025). While remission of T2D is possible, particularly under intensive therapy, significant weight loss, and dietary adherence (Suleiman et al., 2022), it is not a stable state for most patients. Durable restoration of β-cell function, reflected in sustained improvements in glycemic parameters, is associated with early weight reduction of ≥10% in the first years following T2D diagnosis (Morieri et al., 2025). Collectively, these observations underscore the relevance of studying strategies aimed at preserving or restoring the population of insulin-secreting pancreatic cells.

Advances in modern technologies have enabled β-cells to be considered not as a uniform population but as a highly organized and functionally diverse system. Omics platforms, including single-cell level analyses such as single-cell RNA sequencing (scRNA-seq), provide deep insights into β-cell heterogeneity, revealing distinct functional subpopulations that differ in maturity, metabolic activity, and susceptibility to external stressors such as inflammation, glucotoxicity, and the endoplasmic reticulum (ER) stress they mediate. Integration of scRNA-seq with epigenomic approaches, such as single-cell sequencing assay for transposase-accessible chromatin (scATAC-seq) and DNA methylation profiling, allows identification of regulatory elements governing the expression of key genes, including those involved in survival, proliferation, and insulin secretion (Leenders et al., 2024; Rönn et al., 2023). At the same time, increasing attention is being paid to extrapancreatic factors that influence β-cell functionality. Among these, the gut microbiota represents a complex ecosystem capable of modulating metabolism, immune responses, and glucose homeostasis (Hou et al., 2022). Alterations in microbiota composition and functionality have been increasingly linked to T2D pathogenesis (Hrncir, 2022), and microbiota modulation is now being considered as a therapeutic approach (Byndloss et al., 2024). In this context, the development of next-generation probiotics (NGPs) and novel probiotic strategies, such as the use of genetically engineered bacteria designed to express incretin hormones, particularly GLP-1, is of particular interest (Zhang et al., 2024).

The present review aims to highlight the importance of integrating data obtained at the single-cell, tissue, and microbiota levels in order to construct a comprehensive view of the multilevel regulation of β-cells during the progression and treatment of diabetes. In the following sections, we will discuss: (1) β-cell heterogeneity as revealed by scRNA-seq; (2) the role of the gut microbiota in regulating metabolism and β-cell function; and (3) the potential of GLP-1-expressing probiotics as a novel therapeutic strategy. We argue that only an integrative approach combining molecular, cellular, and microbiome-level insights can advance our understanding of diabetes pathogenesis and enable the development of more precise and sustainable therapeutic solutions.

## 2 Molecular and functional heterogeneity of pancreatic β-cells

Pancreatic β-cells were historically considered a homogeneous population, defined primarily by insulin synthesis and secretion in response to hyperglycemia. This paradigm, established through twentieth-century techniques like classical histology, biochemistry and electron microscopy, presumed morphological similarity and uniform functional responses to stimuli among β-cells (Avrahami et al., 2017), and stable expression of key transcription factors such as INS, PDX1, and MAFA (Zhu et al., 2017). However, this view was constrained by methodological limitations. Bulk sequencing approaches, which average gene expression across entire tissues, masked underlying cellular heterogeneity (Chan et al., 2021; Liu X. et al., 2022; Tarifeño-Saldivia et al., 2017). The development of high-resolution single-cell technologies—including scRNA-seq, ATAC-seq, and Patch-seq—has since revealed a landscape of transcriptionally and functionally distinct β-cell subtypes (Camunas-Soler et al., 2020; Geng et al., 2025; Hrovatin et al., 2023; Klein et al., 2025; Olaniru et al., 2023; Tarifeño-Saldivia et al., 2017; Tritschler et al., 2017). Among these, so-called “resistant” β-cells maintain high INS, MAFA, and PDX1 expression under diabetic conditions, preserving function despite hyperglycemia and inflammation (Chong et al., 2024). They are characterized by unique epigenetic profiles and the expression of protective factors. Other subpopulations include quiescent or dedifferentiating cells, proliferatively active cells, as well as those showing resistance to diabetic injury (Aldous et al., 2023).

### 2.1 Functional β-cell subtypes in health and T2D

Recent research has shifted from establishing β-cell heterogeneity to defining the specific functional roles of its subpopulations. In mice, β-cells can be separated into mature (FLTP-high) and immature (FLTP-low) populations using the FLTP (CFAP126) marker. Mature FLTP-high cells are characterized by high PDX1 and insulin content but low proliferative activity, whereas immature FLTP-low cells have a greater proliferative capacity. During the development of insulin resistance, the proportion of FLTP-low cells increases, accompanied by reduced insulin secretion (Bader et al., 2016). A similar maturation gradient exists in humans. Dorrell et al. identified four β-cell subtypes that differ in insulin expression, transcriptomic profiles, and glucose responsiveness. In this context, insulin resistance similarly shifts the balance toward less mature forms, preserving overall cell mass but impairing functional insulin secretion (Dorrell et al., 2016). Further transcriptomic profiling in mice revealed a highly functional subpopulation marked by high CD63 expression. These CD63-high β-cells exhibit active glucose metabolism and enhanced insulin secretion. A similar functional distinction exists in humans: β-cells with high CD63 expression responded rapidly to glucose stimulation and were enriched in genes regulating insulin secretion, while CD63-low cells exhibited reduced functional potential. In type 2 diabetes, both mice and humans show a shift toward CD63-low subpopulations, indicating a progressive loss of functionally mature β-cells (Rubio-Navarro et al., 2023). In humans, a subpopulation of immature β-cells persists at the islet periphery throughout life. These cells produce insulin but are glucose-unresponsive and lack the maturity marker UCN3 (Shrestha et al., 2021). Notably, UCN3 itself, typically a marker of mature β-cells, may appear in fetal islets (Tremmel et al., 2023). Thus, β-cell maturation is a complex and dynamic, reflecting their intrinsic heterogeneity and enduring plasticity.

In addition, genetic factors significantly influence β-cell function. T2D-associated risk alleles (e.g., KCNJ11, SLC30A8, HNF1A) are enriched in regulatory regions of less functional cells β-cells. This suggests that these genetic variantsmay promote a shift toward dysfunctional states (Khetan et al., 2021). Analysis of candidate cis-regulatory elements (cCREs) has shown that some diabetes-associated variants reduce chromatin accessibility at binding sites for transcription factors such as HNF1A and HNF4A, thereby impairing maintenance of a functional phenotype (Wang et al., 2023). Conversely, other variants increase accessibility at sites associated with TCF4, NEUROD1, and NFIA, potentially promoting β-cell dedifferentiation. Dynamic transcriptional responses to stress, mediated by factors like ATF4 and XBP1, also play a central role by balancing adaptive mechanisms with apoptotic pathways (Diane et al., 2024). Disruption of specific factors, such as Taf4 inactivation, leads to loss of β-cell identity and disrupts metabolic and inflammatory pathways (Kleiber et al., 2021).

### 2.2 Stress-responsive and dedifferentiated β-cell states

The characterization of β-cell subpopulations has been advanced by single-nucleus RNA sequencing (snRNA-seq). This technique is suitable for profiling transplanted islets and frozen tissues, where conventional scRNA-seq is unsuitable (Basile et al., 2021; Xie et al., 2024). Applying snRNA-seq, Kang and colleagues identified novel markers for human islet endocrine cells: ZNF385D, TRPM3, LRFN2, PLUT for β-cells; PTPRT, FAP, PDK4, LOXL4 for α-cells; LRFN5, ADARB2, ERBB4, KCNT2 for δ-cells; and CACNA2D3, THSD7A, CNTNAP5, RBFOX3 for γ-cells. By integrating snRNA-seq and scRNA-seq data, the authors also described three β-cell subtypes based on the ratio of mature to immature insulin isoforms: β1 cells with high levels of mature insulin mRNA representing a functionally mature population; β3 cells enriched in insulin pre-mRNA, characterized by reduced maturity or proliferative activity; and β2 cells, which represent an intermediate state. These subtypes functionally specialized: β1 cells were primarily dedicated to insulin secretion, β3 to transcriptional regulation and proliferation, and β2 to metabolism and vesicular transport. Importantly, these subpopulations were also identified in *vivo* transplants, where the proportion of mature β1 cells was higher than *in vitro* cultures, underscoring the role of the physiological microenvironment in maintaining the mature β-cell phenotype (Kang et al., 2023). Interestingly, a study by Weng and colleagues did not identify T2D-specific β-cell subpopulations in their scRNA-seq and snATAC-seq datasets, likely due to inter-individual variation masking disease-associated changes. To address this, they applied a regression-based method (RePACT), which revealed a diabetes-associated epigenetic signature: decreased chromatin accessibility at motifs for HNF1A/B and RFX6, and increased accessibility at motifs for NEUROD1, NFY, and TP53. These changes are consistent with β-cell dedifferentiation and functional decline. Complementary Patch-seq data demonstrated that β-cells expressing HNF1A exhibit reduced Na^+^ influx. This phenotype was linked to the HNF1A target gene FXYD2, a regulator of Na^+^/K^+^-ATPase activity. The loss of HNF1A and FXYD2 may induce membrane hyperpolarization, thereby impairing insulin secretion—a mechanism relevant to both maturity-onset diabetes of the young type 3 (MODY3) and T2D (Weng et al., 2023). Overall, β-cells can be categorized into distinct epigenetic states. Dror and colleagues applied an “epigenetic dosage index” based on cumulative chromatin accessibility to classify β-cells into two groups (Fontcuberta-PiSunyer et al., 2023). The high-index subpopulation showed elevated expression of genes involved in insulin secretion, metabolism, and key transcriptional regulators like PDX1, NKX6.1, and MAFA. In contrast, the low-index subpopulation exhibited reduced transcriptional activity, diminished glucose responsiveness, and features of stress-induced dysfunction. This epigenetic classification proved robust, as similar profiles were observed in both transplanted and diabetic β-cells (Dror et al., 2023).

Although scRNA-seq and snATAC-seq datasets of pancreatic β-cells have not yet been directly integrated with gut microbiome metagenomic or metabolomic profiles within a single unified study, the parallel development of these fields highlights potential points of convergence between β-cell transcriptional states and microbiota-derived metabolites (Son et al., 2020). While direct experimental evidence comparing the responsiveness of specific β-cell subpopulations to SCFAs, GLP-1, or other microbiota-derived metabolites remains limited, existing resources such as HPAP (Kaestner et al., 2019), reveal clear associations between β-cell maturity, chromatin accessibility, metabolic state, and the capacity to sense exogenous signals. Collectively, these observations suggest that β-cell heterogeneity may shape not only the functional responsiveness to incretins but also the overall efficacy of microbiota-targeted interventions aimed at supporting β-cell function. This perspective provides a mechanistic bridge linking β-cell diversity to the broader gut–pancreas axis.

### 2.3 Variability in incretin receptor expression

An important aspect of β-cell heterogeneity is the variability in incretin receptor expression. The incretin axis is a central regulator of glucose homeostasis and a key drug target in type 2 diabetes. Glucagon-like peptide-1 receptor (GLP1R) enhances insulin secretion primarily through cAMP-dependent pathways, promoting insulin exocytosis. The glucose-dependent insulinotropic polypeptide receptor (GIPR) functions similarly but may also be expressed in α- and possibly δ-cells, modulating glucagon and somatostatin secretion (Shilleh et al., 2024). However, scRNA-seq data show that a substantial fraction of β-cells in humans and mice express neither GLP1R nor GIPR, or only one of them (Gray et al., 2020). This contrasts with findings from *Glp1r:mTmG* mouse models, where ∼78% of β-cells appear GLP1R-positive, suggesting that technical limitations or post-transcriptional regulation may explain the discrepancy (Gray et al., 2020). Consistent with this, GLP1R protein is detected in β-cells but not in α- or δ-cells, despite the presence of its mRNA (Gray et al., 2020). The spatial organization of GLP1R within islets may also profoundly affect incretin agonist pharmacodynamics. For instance, Ast et al. demonstrated that GLP1R forms clusters on β-cell membranes, with their distribution and density varying both between and within cells. Furthermore, GLP1R internalization is heterogeneous: different β-cell subpopulations are recruited sequentially, and agonists induce distinct internalization patterns. Exendin-4 triggered internalization in 70%–80% of β-cells, while semaglutide and tirzepatide did so in only ∼50% (Ast et al., 2023). Tong et al. further showed that GLP1R forms nanodomains on β-cell membranes adjacent to α-cells. These β-cells can directly sense glucagon release, and due to prior GLP1R internalization, exhibit a faster and stronger secretory response to glucose than β-cells contacting other β-cells (Tong et al., 2025).

Thus, the modern understanding of β-cells has fundamentally shifted from a view of homogeneity and static function to a model of a highly dynamic and plastic system. This system is composed of distinct subpopulations, which differ in their in their capacity for adaptation, regeneration, and response to extrinsic cues—ranging from pathological signals to therapeutic agents such as incretins and microbiota-derived metabolites. This heterogeneity underpins a dual-therapeutic approach. On one hand, mature, metabolically active subsets (e.g., CD63-high, FLTP-high β-cells) exhibit robust insulin secretion and are prime targets for GLP-1-based therapies, which are known to enhance β-cell function (Salehi et al., 2010). On the other hand, beyond its acute insulinotropic effects, GLP-1 promotes β-cell proliferation and inhibits apoptosis (Liu Y. et al., 2022). Therefore, a complementary regenerative strategy is targeting immature, proliferation-competent, and insulitis-resistant β-cell subsets. This approach aims to maintain or expand this immature pool and guide its maturation to regenerate functional, insulin-secreting β-cells. Consequently, the composition and functional state of β-cell subpopulations are likely key determinants of the efficacy for both microbiota-targeted and incretin-based interventions.

## 3 Plasticity of pancreatic cells: scRNA-seq evidence in diabetes

**FIGURE 1:**
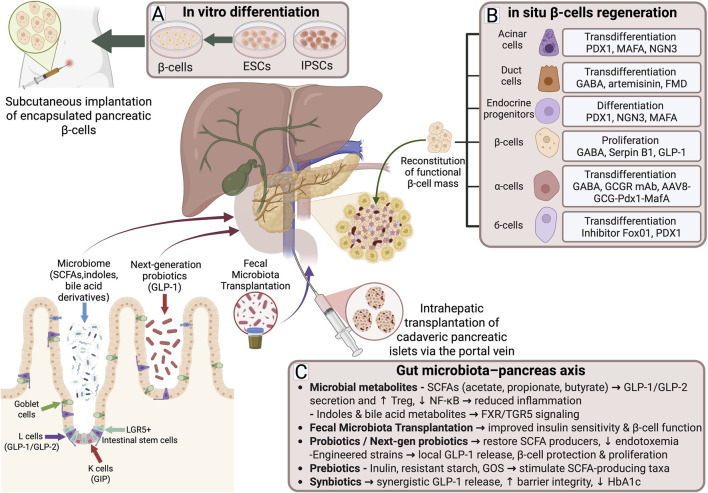
Overview of current strategies for pancreatic β-cell regeneration and modulation in type 2 diabetes. **(A)** *In vitro* approaches include the differentiation of human stem cells into insulin-producing β-cells, with subsequent subcutaneous implantation of encapsulated cells as a potential therapeutic strategy. Intrahepatic transplantation of cadaveric islets *via* the portal vein also represents a clinically applied regenerative approach. **(B)** *In situ* regeneration encompasses transdifferentiation of acinar and ductal cells, differentiation of endocrine progenitors, stimulation of β-cell proliferation, and reprogramming of α- and δ-cells, collectively contributing to the reconstitution of functional β-cell mass. **(C)** The gut microbiota-pancreas axis represents an additional layer of regulation, where microbial metabolites such SCFAs, indoles, and bile acid derivatives enhance incretin secretion, improve insulin sensitivity, and reduce inflammation. Interventions including prebiotcs, synbiotics fecal microbiota transplantation, and next-generation probiotics offer translational opportunities to modulate host metabolism and β-cell function.

Alongside the rapidly advancing *in vitro* approaches, which enable the differentiation of embryonic stem cells (ESCs) or induced pluripotent stem cells (iPSCs) into insulin-producing β-cells for subsequent implantation in encapsulated formats (Pagliuca et al., 2014; Dadheech et al., 2025; Chen et al., 2025; Du S. et al., 2022) (Figure 1A), recent studies highlight complementary *in situ* strategies. In this context, it has been demonstrated that the restoration of β-cell mass can occur not only through proliferation of existing β-cells but also *via* transdifferentiation of other pancreatic cell types, such as α-, δ-, PP-, and ductal epithelial cells (Figure 1B). The plasticity of pancreatic cells, enabling them to acquire β-cell identity, is considered a potential therapeutic strategy for diabetes, either through activation of endogenous regenerative mechanisms or through the generation of functionally mature β-cells under controlled *in vitro* conditions, as we have detailed in our previous review (Chernysheva et al., 2024). Pancreatic endocrine cells are generally considered terminally differentiated; however, substantial evidence indicates that they retain significant plasticity under stress or genetic manipulation (Ediger et al., 2017; Gutiérrez et al., 2017; Saleh et al., 2021; Thorel et al., 2010). The most extensively studied source is α-cells. Following near-total ablation of β-cells in mice, α-cells can transform into bihormonal (insulin^+^/glucagon^+^) cells and subsequently into monohormonal insulin^+^ cells (Thorel et al., 2010). Key regulators of this plasticity include the genes Arx and Dnmt1: their suppression in mice reprograms α-cells into functional β-like cells, though with limited secretory capacity (Chakravarthy et al., 2017; Courtney et al., 2013; Matsuoka et al., 2017; Xiao et al., 2018; Zhang et al., 2016). An alternative approach involves Pax4 overexpression, which induces β-cell neogenesis from α-cells and corrects diabetes in animal models (Collombat et al., 2009). Interestingly, Pax4 knockout promotes the transdifferentiation of ε-cells (ghrelin^+^) into β-cells in zebrafish (Yu et al., 2023). However, these mechanisms are context-dependent and not always reproducible. For instance, Feng et al. (2020), using an streptozotocin (STZ)-induced diabetes model, did not detect a direct α to β-cell transition (Feng et al., 2020).

Acinar and ductal pancreatic cells, as well as cells from other organs, may also serve as targets for reprogramming. In a recent study, Dahiya et al. (2024) demonstrated that inhibition of focal adhesion kinase (FAK) in adult mice *in vivo* induced partial transdifferentiation of acinar cells located near islets into β-like cells, which subsequently integrated into islets and improved glycemia in diabetic mice (Dahiya et al., 2024). Another described pathway involved the transdifferentiation of cells into insulin-producing β-like cells with concomitant glucose normalization, as reported by Du W. et al. in an STZ-induced diabetes model in mice under triple blockade of FoxO1, Notch, and Tgf-β (Du et al., 2022). Remarkably, they observed that the Paneth/goblet lineage, intestinal enteroendocrine cells (EECs), can also undergo conversion into the insulin lineage. A well-known combination of factors - Pdx1, Ngn3, and MafA (PNM) has been shown to convert exocrine cells into insulin-producing β-like cells, ameliorating hyperglycemia in mice (Lee et al., 2013; Zhou et al., 2008). This strategy has also proven effective for Sox9^+^ ductal liver murine cells (Banga et al., 2014) and intestinal cells (Chen et al., 2014). However, hyperglycemia suppresses PNM-mediated acinar cell transdifferentiation, underscoring the importance of metabolic control (Cavelti-Weder et al., 2016). Human exocrine pancreatic cells can likewise respond to pro-endocrine stimuli: reprogramming human α- and γ-cells with PDX1/MAFA generates functional insulin^+^ cells that, upon transplantation, improve glycemia in mice (Furuyama et al., 2019). A similar effect was observed in human acinar cells transduced directly with lentiviruses expressing MAPK and STAT3 *in vitro* (Lemper et al., 2015). In the study by Fontcuberta-PiSunyer et al., human fibroblasts were directly reprogrammed using adenoviral vectors delivering NEUROG3, PDX1, and MAFA, followed sequentially by PAX4 and NKX2-2. Notably, PAX4 and NKX2-2 enhanced the yield of β-like cells compared to PNM treatment alone (Fontcuberta-PiSunyer et al., 2023).

Transdifferentiation into β-cells is possible from α-, ε-, acinar, ductal, intestinal EECs, and even fibroblasts, *via* regulation of key transcription factors including PNM, PAX4, and Arx/Dnmt1. While pancreatic cell plasticity offers promising avenues for restoring β-cell mass in diabetes through reprogramming of endogenous cells or transplantation of induced β-cells generated *in vitro*, the efficiency and applicability of these processes in humans remain under active investigation. Despite significant successes in animal models, clinical translation is limited by the functional immaturity of reprogrammed cells. Therefore, particular interest lies in elucidating the molecular and functional changes occurring in β-cells and other islet endocrine cells in T2D, as these cellular adaptations and dysfunctions constitute the foundation of disease pathogenesis and provide a rationale for the development of targeted therapeutic strategies. Before examining microbiota-derived influences, it is important to outline the intrinsic transcriptional and epigenetic disturbances that characterize β-cell dysfunction in T2D. These molecular alterations define the baseline state of β-cells and other islet endocrine cells and shape their responsiveness to external regulatory signals.

### 3.1 Transcriptomic alterations in islet cells in T2D

In T2D, pancreatic β-cells exhibit characteristic molecular impairments, including reduced transcriptional activity of genes involved in insulin secretion. Chromatin accessibility is altered in regulatory regions controlled by key transcription factors such as MAFA and PDX1. In addition, destabilization of the spatial architecture of the islets of Langerhans and disruptions in intercellular communication are observed, particularly in the context of enhanced pro-inflammatory signaling and remodeling of the microenvironment (Karakose et al., 2024). In recent years, an increasing number of previously unknown molecular markers specific to distinct islet cell types have been described. For example, in γ-cells: SERTM1, ABCC9, GHRL, and GHRLOS; in ε-cells: NPY1R, OPRK1, and PTGER4. Moreover, subtype-specific expression of transcription factors (TFs) has been demonstrated: MAFA and SIX2/3 in β-cells, IRX2 in α-cells, NKX6-3 in δ-cells, MEIS2 in γ-cells, and AFF3 in ε-cells. In the study by Segerstolpe, T2D donors exhibited reduced β-cell numbers and decreased INS expression, as well as pronounced suppression of FXYD2, a Na,K-ATPase gene associated with β-cell function. Conversely, expression of GPD2 and LEPROTL1, genes linked to metabolic stress and reduced leptin sensitivity, was increased (Segerstolpe et al., 2016). Overall, T2D is accompanied by complex dysregulation of gene expression and epigenetic status, directly impacting β-cell viability and insulin secretion. For instance, Bacos et al. reported that over 85% of the 395 identified differentially expressed genes (DEGs) in islets from T2D donors were associated with regions of open chromatin or DNA methylation, with some overlapping T2D-associated single nucleotide polymorphisms (SNPs). Functional experiments further showed that overexpression of PAX5, one of the genes upregulated in T2D, led to reduced insulin secretion, suppression of mitochondrial respiration, and disruption of energy balance through regulation of downstream genes such as BARX1, NEFL, PCOLCE2, and SFRP1 (Bacos et al., 2023). In the study by Elgamal et al., 84 genes showed significant expression changes, the vast majority (n = 79) localized to β-cells. The most strongly upregulated genes included TSHR, SLC4A4, and TNFRSF11B, associated with hormonal signaling and growth regulation. These genes were functionally enriched in RNA-processing and hormone-response pathways. Conversely, downregulation was observed in genes linked to mitochondrial function and antioxidant defense, including ASCL1, located near genetic loci of T2D risk (Elgamal et al., 2023). Furthermore, patients with T2D display activation of NFE2L2, a key regulator of the oxidative stress response. Its expression was elevated in β-cells of diabetic patients, particularly in immature and senescent subtypes, along with its targets (GSTA4, GSTM3, GCLC, CYP2R1). This activation occurs not only as a response to stress but also under conditions of impaired autophagy *via* an alternative SQSTM1 p62-mediated pathway, the levels of which are also elevated (Marques et al., 2022).

Some studies have focused on the dynamics of the islet transcriptomic response to glucose. Grenko et al. demonstrated that glucose exposure induces an active and sustained transcriptional response predominantly in β-cells, peaking at 8 h and persisting up to 24 h. Importantly, this study identified novel candidate genes linked to insulin secretion and T2D risk: ERO1B (ER stress and protein folding), HNRNPA2B1 (an RNA-binding protein regulating insulin levels), and RHOBTB3 (involved in secretory protein trafficking), whose inhibition reduced intracellular insulin (Grenko et al., 2024). Taken together, these findings show that in diabetes, β-cells are altered both quantitatively and qualitatively, forming subpopulations with varying degrees of stress response and functional vulnerability. The use of scRNA-seq has revealed previously unrecognized heterogeneity among β-cells and their differentiated responses to metabolic and inflammatory signals. The progression of T2D is driven not only by β-cell dysfunction but also by intrinsic vulnerability factors within pancreatic cells themselves, manifested in transcriptomic alterations, as well as complex intercellular interactions involving immune, ductal, acinar, and stromal cells of the pancreas. These insights deepen our understanding of diabetes pathogenesis and highlight the need for therapeutic strategies capable of selectively targeting both vulnerable and resilient populations of β-cells and other pancreatic cell types. In this regard, a particularly promising paradigm is the modulation of the gut microbiome as a regulator of metabolism and β-cell function, which will be discussed in the following section. These findings deepen our understanding of diabetes pathogenesis and highlight the need for therapeutic strategies capable of selectively targeting both vulnerable and resilient populations of β-cells and other pancreatic cell types.

## 4 Pancreas–microbiota interactions

The application of multi-omics approaches to the study of the pancreas provides the foundation for developing personalized therapeutic strategies tailored to the molecular and cellular profiles of individual patients (Mou et al., 2025). One of the most ambitious initiatives in this field is the HPAP, which aims to comprehensively map the molecular and morphological alterations of the pancreas in T2D. Within this initiative, a standardized protocol is employed for the collection and analysis of pancreatic tissue from donors with normoglycemia, prediabetes, and overt T2D. A distinctive feature of HPAP is its multi-omics design, integrating snRNA-seq, snATAC-seq, whole-genome bisulfite sequencing (WGBS), morphometric techniques, and spatial technologies (Visium, CODEX). This approach has revealed pronounced spatial heterogeneity in the expression of INS and other key genes, not only between donors but also across different pancreatic lobes and even between individual islets within the same anatomical region. Such findings emphasize the necessity of spatial analysis when interpreting phenotypic variation. Particular attention is devoted to β-cell heterogeneity and dysfunction, including reduced expression of INS, MAFA, and genes regulating insulin secretion, alongside activation of stress-associated programs (ATF4, DDIT3). Beyond transcriptional changes, epigenetic modifications have also been identified, including altered DNA methylation in regulatory regions associated with diabetes. Spatial methods have further demonstrated that islet dysfunction and the composition of their cellular niche vary according to anatomical location. Additional findings describe alterations in immune, stromal, and acinar cells, whose interactions with endocrine elements may substantially influence T2D pathogenesis. Another important direction has been the study of the islet secretome, which provides opportunities for identifying novel biomarkers and potential therapeutic targets (Shapira et al., 2022). While these pancreatic multi-omics resources offer unprecedented resolution of intra-organ heterogeneity, they remain focused almost exclusively on the pancreas and currently do not include matched profiles of the gut microbiome, despite the growing recognition of the gut–pancreas axis in metabolic disease.

Collectively, these findings confirm that β-cell dysfunction results from the cumulative impact of chronic metabolic stressors, including hyperglycemia, lipotoxicity, and inflammation (Blüher et al., 2023). Crucially, the interaction between stress-induced regulatory programs and genetic predisposition determines the adaptive capacity of β-cells under the acute physiological pressures characteristic of diabetes. In the study by Sokolowski et al., pathological states such as ER stress and inflammation were modeled, enabling the identification of context-dependent regulatory networks and their association with T2D-linked loci. The authors demonstrated that activation of genes involved in the unfolded protein response (UPR) is specific to ER stress, whereas pro-inflammatory cascades are initiated exclusively by cytokine stimuli. Moreover, T2D-associated GWAS loci, including STARD10, were shown to colocalize with differentially accessible chromatin regions (DARs) whose accessibility was altered by stress conditions. High-throughput chromatin conformation capture with immuno precipitation (HiChIP) analysis further uncovered novel stress-dependent interactions between enhancers and candidate genes, including ATF6B, thereby clarifying their functional relevance. Key transcription factors activated in response to ER stress and inflammation (XBP1, CHOP, STAT1) were also identified, highlighting potential avenues for targeted interventions (Sokolowski et al., 2024). In addition to metabolite-driven pathways, multi-omics evidence suggests that microbial dysbiosis may influence β-cell states *via* LPS-dependent inflammatory signaling. Studies in metabolic disease consistently report elevated systemic LPS levels associated with dysbiosis and metabolic endotoxemia (Dong et al., 2022), and these increases correlate with β-cell transcriptional stress signatures observed in scRNA-seq analyses, including the expansion of ATF4^+^ and XBP1^+^ subclusters and the downregulation of maturity-related transcription factors such as PDX1 and MAFA under inflammatory conditions (Sokolowski et al., 2024). Such stress-associated β-cell states are known to display impaired incretin responsiveness due to reduced GLP1R signaling and diminished cAMP pathway activation (Luo et al., 2013)[Gao], suggesting that inflammatory axes linked to dysbiosis may differentially affect vulnerable β-cell subpopulations. Incorporating LPS-associated microbial signatures into β-cell single-cell analyses may therefore help elucidate how microbiota-derived inflammatory cues intersect with established β-cell heterogeneity.

An example of the microbiota–host axis involves the microbiota–tryptophan–AHR–GLP-1 pathway. Microbial conversion of tryptophan into indole-derived metabolites activates AHR signaling in enteroendocrine L-cells, thereby enhancing GLP-1 secretion (Chimerel et al., 2014). In db/db mice, dapagliflozin reshapes the gut microbiota and redirects tryptophan metabolism toward increased L-tryptophan–derived metabolites, leading to augmented GLP-1 production, higher circulating GLP-1 and C-peptide levels, and expansion of β-cell mass *via* enhanced proliferation; these effects are largely abrogated by GLP-1R blockade. Although this work does not resolve β-cell subpopulations at single-cell resolution, it offers mechanistic evidence that microbiota-dependent tryptophan metabolites can promote β-cell regeneration through a gut microbiota–tryptophan–GLP-1–GLP-1R axis (Jiang et al., 2024). GLP-1–responsive β-cells exhibit enhanced glucose-stimulated insulin secretion through activation of the cAMP-dependent signaling cascade: ligand binding to GLP-1R stimulates adenylyl cyclase, elevates intracellular cAMP levels, and sequentially engages both PKA- and Epac2-mediated pathways, thereby promoting insulin-granule exocytosis and increasing the secretory competence of β-cells (Luo et al., 2013). Parallel studies in other organ systems have shown that integrating microbial composition with circulating metabolite profiles and host gene expression can reveal causal gut–host pathways. These approaches provide a conceptual framework for future studies aiming to link gut microbiota–derived signals with β-cell biology.

While pancreatic studies have primarily focused on cellular and molecular heterogeneity, a large proportion of microbiome research has relied on correlational associations between microbial composition and disease development. This reliance significantly complicates the establishment of causal mechanisms and limits the translational potential of these findings. To overcome these barriers, a strategy has been proposed that integrates multi-omics data with multicenter cohort studies consolidated into meta-cohorts, followed by stepwise validation using *in silico*, *in vitro*, *ex vivo*, and *in vivo* models. This approach refines observed associations, confirms their mechanistic plausibility, and identifies the most promising targets for preclinical and clinical testing (Turjeman et al., 2025).

In summary, the systemic integration of pancreatic data underscores the effectiveness of multi-omics approaches in unraveling the complex interactions among cellular, genetic, and epigenetic factors. A logical extension of this framework is the consideration of another key regulatory system -the gut microbiota, which also makes a substantial contribution to diabetes pathogenesis and interacts with the islet apparatus within the framework of the “gut–pancreas” axis. Future studies combining β-cell states defined by scRNA-seq and snATAC-seq with quantitative measures of SCFA, indole derivatives, or endotoxemia have the potential to reveal whether specific β-cell subpopulations preferentially associate with distinct microbiota-derived signatures or display differential transcriptional responses to incretin- and SCFA-dependent interventions, thereby advancing a systems-level understanding of microbiota–β-cell interactions.

## 5 Gut microbiota as a regulator of metabolism and β-cell function

Contemporary studies confirm the close interconnection between the gut and the pancreatic islet apparatus, commonly referred to as the “gut–islet axis” (Marroncini et al., 2024) (Figure 1C). Intestinal EECs secrete a broad spectrum of hormones that regulate glucose levels and stimulate insulin secretion by β-cells. Among them, L-cells, which release GLP-1, K-cells, which produce GIP, and I-cells, which secrete cholecystokinin, are considered key. Although EECs comprise only about 1% of the intestinal epithelium, their contribution to the regulation of glucose and energy metabolism is highly significant (Solcia et al., 1978). The contribution of incretins to insulin secretion is particularly remarkable: in healthy individuals, GLP-1 and GIP account for up to 60% of postprandial insulin secretion (Salehi et al., 2010).

### 5.1 Microbial production of SCFAs and their systemic effects

**FIGURE 2:**
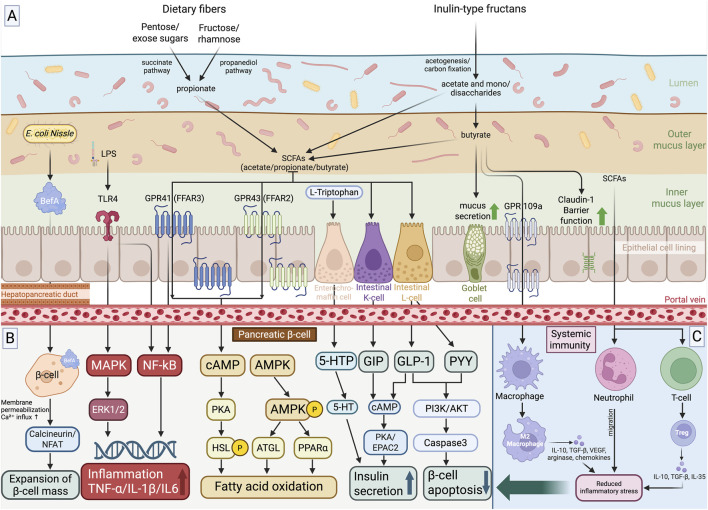
Overview of Gut Microbiota–Derived Metabolite Signaling Toward the Pancreatic β-Cell. **(A)** Dietary substrates: pentoses, hexoses and inulin-type fructans are fermented by the gut microbiota to produce short-chain fatty acids (SCFAs) *via* the succinate and propanediol pathways; butyrate is additionally generated during the degradation of inulin-like fructans. **(B)** The bacterial protein BefA in β-cells increases membrane permeability to Ca^2+^ and activates the calcineurin/NFAT pathway, thereby enhancing β-cell neogenesis and proliferation, whereas LPS from Gram-negative bacteria signals *via* TLR4 to trigger MAPK/ERK1/2- and NF-κB-dependent inflammatory cascades. Microbial tryptophan metabolites stimulate enterochromaffin, K- and L-cells, increasing the production of 5-HTP/5-HT, GIP and GLP-1, which reach the pancreas through the portal circulation, activate GIPR and GLP1R on β-cells, raise intracellular cAMP and engage PKA- and EPAC2-dependent cascades, thereby potentiating insulin granule exocytosis and glucose responsiveness; GLP-1 additionally activates PI3K/AKT signaling and reduces caspase-3–mediated apoptosis. Butyrate, acting *via* GPR109A on goblet cells, stimulates mucus secretion and claudin-1 expression, thereby strengthening the epithelial barrier. **(C)** SCFAs and other microbial metabolites polarize macrophages toward an M2-like phenotype and support Treg differentiation, accompanied by increased secretion of IL-10 and TGF-β, reduced systemic inflammation, and indirect protection of β-cells. Created in BioRender. Ruchko, E. (2025) https://BioRender.com/3soms7k.

One of the critical factors influencing the function of this system is the gut microbiota - a phylogenetically established community of microorganisms inhabiting the gastrointestinal tract. Normal gut microbiota is subdivided into obligate (about 90%), facultative (9.5%), and transient (0.5%) populations (Smolinska et al., 2017). More than 90% of the microbiota in the distal colon of healthy individuals is represented by *Bacteroidetes* and *Firmicutes*: the former predominantly Gram-negative bacteria, and the latter mainly Gram-positive, such as lactobacilli (Silva et al., 2016). The remaining ∼10% comprises Proteobacteria (including *Escherichia* and *Helicobacter*), *Actinobacteria*, *Fusobacteria*, *Verrucomicrobia*, *Cyanobacteria*, and *Spirochaetes*, as well as fungi, protozoa, and viruses. This complex microbiocenosis dynamically responds to the physiological state of the host and to pathological processes, undergoing both quantitative and qualitative shifts. The primary nutritional substrate for the microbiota consists of undigested dietary residues, primarily starches and polysaccharides resistant to enzymatic hydrolysis. Their fermentation by bacteria leads to the formation of short-chain fatty acids (SCFAs): acetate (C2), propionate (C3), butyrate (C4), and, to a lesser extent, formate (C1) and valerates (C5) (Kim et al., 2014). Acetate and propionate are mainly synthesized by *Bacteroidetes*, while butyrate is produced by *Firmicutes* (Baothman et al., 2016). SCFAs exert diverse effects on the host: they strengthen intestinal barrier and immune functions, normalize body weight, glucose levels, and insulin sensitivity (Baothman et al., 2016). SCFAs are known to suppress NF-κB activation and reduce the production of pro-inflammatory cytokines (Kim et al., 2014). Butyrate, in particular, with its potent immunomodulatory properties, inhibits histone deacetylases in dendritic cells and macrophages and activates G-protein-coupled receptors (GPCRs), leading to reduced production of IL-12 and IL-6 and the promotion of regulatory T-cell activation. Propionate exerts similar effects, whereas acetate demonstrates a dual role: in some contexts, it may stimulate the production of pro-inflammatory cytokines and enhance inflammation (Smolinska et al., 2017). Figure 2A outlines the upstream metabolic layer, showing how dietary pentoses, hexoses and inulin-type fructans are fermented into SCFAs (including butyrate) with concurrent reinforcement of epithelial barrier integrity, whereas Figure 2C depicts the immunological arm, in which SCFAs and related metabolites drive M2-like macrophage polarization and Treg differentiation, increase IL-10/TGF-β production and thereby attenuate systemic and islet inflammation to indirectly preserve β-cell function.

The mechanisms of SCFA action include histone deacetylase inhibition, which alters gene expression, and activation of the GPR41 and GPR43 receptors on intestinal L-cells. This stimulates the secretion of GLP-1 and GLP-2 and indirectly affects β-cells and adipocytes by regulating insulin secretion, inflammatory processes, and lipid metabolism (You et al., 2022). In addition, SCFAs activate GPR43/FFAR2 (Tolhurst et al., 2012) and GPR41/FFAR3 (Priyadarshini and Layden, 2015), which also enhance incretin secretion. Notably, GPR41/FFAR3, coupled to Gαi proteins, significantly increases GLP-1 secretion when stimulated with the selective agonist AR420626 (Nøhr et al., 2013), opening opportunities for pharmacological regulation of this pathway. Interestingly, the suppression of GLP-1 secretion by ketone bodies, such as β-hydroxybutyrate, was shown to be sensitive to pertussis toxin, implicating the involvement of SCFA receptors, including GPR41/FFAR3 (Wallenius et al., 2020). An additional contribution to incretin regulation is made by GPR119, which is activated by monoacylglycerols generated during triglyceride hydrolysis. It has been demonstrated that co-activation of GPR119 and GPR40/FFAR1 on EECs markedly enhances GLP-1 release in primary cell cultures (Ekberg et al., 2016). Table 1 provides a comparative overview of microbiota-based interventions in diabetes, emphasizing their underlying mechanisms and reported effects on β-cell function. Figure 2B focuses on direct and incretin-mediated signaling to the β-cell, depicting how BefA, LPS and microbial tryptophan metabolites act on intestinal epithelial and enteroendocrine cells to modulate GIP and GLP-1 secretion and how these hormones, together with BefA, engage GIPR/GLP1R, cAMP–PKA/EPAC2 and PI3K/AKT pathways to regulate β-cell Ca^2+^ influx, survival, proliferation and insulin granule exocytosis.

**TABLE 1:** Comparative overview of microbiota-based interventions in diabetes: mechanisms and β-Cell effects.

Strategy	Mechanism of action	Examples of interventions	Effects on β-cells and the organism	Advantages	Limitations and safety
Probiotics	Colonization of the GI tract with beneficial strains, immune modulation, stimulation of GLP-1/GLP-2, reduction of inflammation	*Bifidobacterium longum*, *Akkermansia muciniphila*, *Roseburia intestinalis*	Increased GLP-1/PYY secretion, reduced inflammation, potential improvement in HOMA-β; enhanced insulin sensitivity, insulin secretion, and reduced glycemia	Safety (GRAS), availability	Strain/dose heterogeneity; effect depends on baseline microbiota
Prebiotics	Stimulation of SCFA-producing bacteria growth, improvement of gut barrier function	Inulin, galactooligosaccharides, resistant starch	Increased GLP-1 secretion *via* FFAR2/3 on L-cells; maintenance of an anti-inflammatory milieu for β-cells; reduction in HbA_1_c, increase in SCFAs, improvement in HOMA-IR	Availability, dietary compatibility	Flatulence/dyspepsia, dose-dependency, heterogeneous effects depending on dose/duration and concomitant therapy
Synbiotics	Combination of probiotic and prebiotic for synergistic effect	*Lactobacillus plantarum* + inulin	Increased GLP-1 secretion, reduced inflammation, improved glycemia	Potential synergy	Requires matching of “strain–substrate” pairs
Postbiotics	Direct administration of bacterial metabolites (SCFAs, indoles, TMA inhibitors)	Butyrate, propionate, indole-3-propionate	Direct increase in GLP-1 secretion *via* SCFAs; reduced inflammation, immune modulation, improved β-cell function	Safety, reproducibility	Limited availability/formulations
Fecal Microbiota Transplantation (FMT)	Transfer of microbiota from healthy donor; restoration of microbiome balance	FMT from metabolically healthy donors	Indirect improvement of β-cell function through reduced inflammation and enhanced incretin secretion; improved insulin sensitivity; prediabetes remission	Strong lever of change	Donor selection, regulatory requirements; heterogeneous outcomes depending on donor/recipient composition, administration mode, and concomitant therapy
Dietary Interventions	Modulation of microbiota composition through nutrition	Mediterranean diet, ketogenic diet, low-glycemic index diet	Reduced endotoxemia, indirect β-cell protection, increased SCFA secretion, reduced inflammation, improved glucose metabolism	High applicability	Patient adherence, individual variability
Antibiotics	Selective or broad suppression of pathogenic microbiota	Rifaximin, vancomycin	Temporary improvement in insulin sensitivity	Experimental tool for studying microbiota–metabolism interactions	Dysbiosis, resistance, unpredictable effects, risks for gut epithelial barrier and β-cells
NGPs (Next-Generation Probiotics)	Use of genetically engineered strains to synthesize therapeutic molecules	*Lactobacillus reuteri* D8, *E. coli* Nissle–GLP-1	Local delivery of hormones and peptides, improved glycemia	Targeted action, “bioreactor” in the gut	GMO regulation, control of gene construct expression
Bile Acid Modulators	FXR/TGR5-mediated crosstalk between microbiota and host	Colesevelam, obeticholic acid, ASBT inhibitors, probiotics/enzymatic approaches influencing BSH	Enhanced GLP-1 secretion *via* TGR5; improved glycemic control, lipid profile, and insulin sensitivity through FXR/TGR5 activation and modulation of bile acid/microbiota pools	Additional pathway complementing SCFA mechanism	Pleiotropic effects
Phages/Anti-colonization Approaches	Targeted suppression of pathogens	Bacteriophage cocktails	Selective elimination of pathobionts or unfavorable bacteria; reduced endotoxemia and inflammation; improved glucose tolerance	Specificity	Evolution of resistance

### 5.2 Microbial factors directly modulating β-cells: LPS, indoles and BefA

SCFAs, particularly butyrate, protect β-cells not only by enhancing GLP-1 secretion but also through profound immunomodulatory effects that attenuate islet inflammation. Butyrate functions as a potent histone deacetylase inhibitor, thereby shifting dendritic cells and macrophages toward a tolerogenic, anti-inflammatory phenotype (Chang et al., 2014; Ji et al., 2016). In dendritic cells, histone deacetylase inhibition suppresses IL-12 and IL-6 production while increasing IL-10 synthesis, promoting the expansion of Foxp3^+^ regulatory T cells (Treg). The resulting increase in Treg abundance dampens metaflammation and reduces circulating and islet levels of TNF-α, IL-1β, and IFN-γ, which in turn alleviates β-cell ER stress and protects against cytokine-induced apoptosis (Kaisar et al., 2017). BefA is a secreted bacterial protein identified in intestinal commensals belonging to the Aeromonadaceae family and certain *Proteobacteria*. Preclinical studies demonstrate that BefA increases β-cell mass by promoting both neogenesis from endocrine progenitors and proliferation of mature β-cells (Hill et al., 2022). At the molecular level, BefA is associated with activation of Notch- and ERK-dependent signaling, two pathways central to cell-cycle regulation and maintenance of endocrine progenitor pools. Notch activation supports the persistence of immature β-cell precursors and enhances their likelihood of differentiating into β-cells, while ERK signaling promotes proliferation of existing mature β-cells. Together, these pathways generate a “proliferative bias,” shifting the β-cell compartment toward expansion. Importantly, BefA-mediated effects are independent of inflammatory cascades: administration of purified BefA or conditioned media enriched in BefA does not elevate pro-inflammatory cytokines and does not activate NF-κB, JNK, or STAT1 signaling. Collectively, BefA represents a notable example of a microbiota-derived factor capable of directly enhancing β-cell plasticity, expanding β-cell mass, and supporting restoration of functional β-cell reserves.

## 6 Mechanisms of dysbiosis-induced effects on insulin resistance and β-cell function

Alterations in the composition and functional activity of the gut microbiota (dysbiosis) are considered a significant factor in the pathogenesis of both T2D and type 1 diabetes (T1D). The transfer of microbiota from obese donors to recipient mice induced the development of hyperglycemia and hyperinsulinemia, confirming the direct influence of the gut microbiome on pancreatic β-cells (Martínez-Montoro et al., 2022). Early studies, including the TEDDY project, demonstrated that the microbiota of children with T1D differs substantially from that of healthy peers. In controls, Lactococcus lactis, *Streptococcus* thermophilus, and other SCFA-producing species predominated, whereas children with T1D showed an increased abundance of *Roseburia hominis*, *Alistipes shahii*, and *Bifidobacterium pseudocatenulatum*. These changes were accompanied by inflammatory alterations in the intestinal mucosa and elevated levels of lipopolysaccharides (LPS), which activate TLR4-dependent inflammatory pathways and contribute to β-cell damage (Vatanen et al., 2018). Moreover, serum LPS concentrations correlate with the severity of insulin resistance in humans (Lassenius et al., 2016; Moreira et al., 2015).

In T2D, dysbiosis is also associated with the development of insulin resistance. Patients typically present with reduced numbers of *Faecalibacterium*, *Roseburia*, *Akkermansia*, and *Bifidobacterium*, alongside an increased abundance of *Ruminococcus*, *Fusobacteria*, and *Blautia*. Species-level diversity is particularly important: for example, *F. prausnitzii* and *R. intestinalis* are associated with metabolic health, whereas *R. inulinivorans* is more frequently identified in diabetes (Bielka et al., 2022). In preclinical models, there is already evidence indirectly supporting a differential β-cell response to microbiota-derived signals. As summarized by Martínez-Montoro et al., modulation of the gut microbiota in mice (*via* antibiotics, FMT, or dietary interventions) alters the profiles of SCFAs, LPS, and bile acids, and this is accompanied by a shift in the balance between compensatory β-cell hyperfunction and subsequent exhaustion: animals harboring an “obesogenic” microbiota exhibit early hyperinsulinemia, hypersecretion, and β-cell hypertrophy, whereas microbiota normalization reduces adipose tissue inflammation, improves insulin sensitivity, and partially restores β-cell function (Martínez-Montoro et al., 2022). These findings support the concept that the microbiota indirectly modulates β-cell subpopulations through the “adipose tissue–inflammation–insulin resistance” and “SCFA/LPS–incretins–β-cells” axes, even though direct single-cell studies demonstrating differential responses of individual β-cell subtypes to microbiota-derived metabolites are not yet available (Doumatey et al., 2020). The translocation of microbial components into the systemic circulation activates the innate immune response and promotes the development of chronic metaflammation (Huda et al., 2021). Interaction of LPS with TLR4 on the surface of adipocytes and macrophages triggers signaling cascades *via* the MyD88 or TRIF adaptors, leading to NF-κB activation and induction of pro-inflammatory genes encoding IL-1β, IL-6, and TNF-α (Cani et al., 2007; Lumeng, 2013). These signals drive serine phosphorylation of the insulin receptor substrate IRS1, thereby blocking insulin signal transduction and contributing to insulin resistance (Choe et al., 2016; McLaughlin et al., 2017). Systemic inflammation in T2D is further exacerbated by reduced levels of bacteria with anti-inflammatory activity that stimulate IL-10 and IL-22 secretion (*Roseburia*, *Akkermansia*, *Bacteroides*, *Faecalibacterium*), as well as those inducing Treg cells (*Clostridium clusters* IV and XIVa). Simultaneously, the proportion of pro-inflammatory species (*Ruminococcus gnavus*, *Fusobacterium nucleatum*, *Prevotella copri*) increases, enhancing pro-inflammatory cytokine secretion and reducing SCFA production along with tryptophan-derived immunomodulatory metabolites, thereby promoting insulin resistance (Huda et al., 2021).

Functional features of the microbiota also play a key role. Metagenomic and metabolomic studies have shown that in humans and mice with insulin resistance, microbial pathways involved in the breakdown of complex carbohydrates (trehalose, maltose, fructose) are enriched. Transplantation of such microbiota into germ-free mice decreased their insulin sensitivity, whereas inhibition of key enzymes in carbohydrate metabolism restored it (Takeuchi et al., 2023). Increasing attention is also directed toward mechanisms of direct microbial influence on host cells. A striking example is the microbial protein BefA, which can enter the bloodstream and stimulate β-cell proliferation or neogenesis without inducing inflammation. BefA increased β-cell mass during development and in adult animals, improving glycemic control in diabetic mice, thus pointing to the possibility of direct modulation of β-cell plasticity by the microbiota (Hill et al., 2022).

Comprehensive approaches integrating multi-omics data (metagenomics, metabolomics, transcriptomics) have revealed associations between the microbiota, β-cell function, and metabolic status. In the study by Yao et al., systemic integration of patient data with varying levels of glycemic control showed that SCFA-producing *Faecalibacterium*, *Roseburia*, and *Akkermansia* were associated with improved insulin secretion and reduced glucose levels, whereas an abundance of *Ruminococcus* gnavus and *Escherichia-Shigella* correlated with hyperglycemia and impaired β-cell activity (Yao et al., 2024). Modern methods of 16S rRNA sequencing and metagenomic analysis provide high-resolution insights into the microbiota, allowing the identification of specific profiles associated with diabetes and enabling the development of personalized dysbiosis-correction strategies. Beyond its immunomodulatory effects, the interaction of the microbiota with the immune system is now recognized as an important regulator of energy balance, as well as glucose and lipid metabolism, exerting systemic effects on the host (Mederle et al., 2024).

Thus, the gut microbiota serves as a critical regulator of metabolic processes and β-cell function. Its alterations in diabetes are regarded not only as markers of T1D and T2D pathogenesis but also as active factors shaping disease progression. In this context, microbiota modification represents a promising strategy for metabolic regulation and the preservation of β-cell functional activity.

### 6.1 Mechanistic pathways linking dysbiosis to β-cell dysfunction

Intestinal dysbiosis influences β-cell function through several interconnected axes, including inflammatory, metabolic, and incretin-related pathways. One of the central mechanisms involves increased translocation of LPS into the systemic circulation as a consequence of impaired intestinal barrier integrity. LPS activates the TLR4 receptor on macrophages, dendritic cells, and adipocytes, triggering NF-κB–dependent production of pro-inflammatory cytokines such as TNF-α, IL-1β, and IL-6 (Van Der Bruggen et al., 1999; Wu et al., 2009). These circulating cytokines reach the pancreas and intensify stress responses within β-cells—most notably ER stress and activation of the unfolded protein response—leading to reduced expression of key β-cell maturity factors, including MAFA and PDX1, suppression of insulin gene transcription, and impaired secretory function (Nordmann et al., 2017). Chronic low-grade inflammation simultaneously promotes the development of peripheral insulin resistance. Under conditions of insulin resistance, β-cells are compelled to maintain glycemic control through compensatory hypersecretion of insulin. Sustained secretory demand leads to intracellular Ca^2+^ overload, mitochondrial dysfunction, heightened oxidative stress, and progressive β-cell exhaustion (Dinić et al., 2022). At this stage, β-cells lose their adaptive hyperfunctional capacity and transition toward dysfunction and apoptosis. Figure 2C highlights the immunological arm of this axis, showing how SCFAs and related metabolites polarize macrophages toward an M2-like phenotype, promote Treg differentiation and increase IL-10/TGF-β production, thereby dampening systemic and islet inflammation and indirectly preserving β-cell function.

An important feature of dysbiosis is the reduction of butyrate-producing taxa such as *Faecalibacterium prausnitzii* and *Roseburia* spp. (Huang et al., 2025; Xuan et al., 2023). The resulting decline in SCFAs, particularly butyrate, diminishes activation of FFAR2/FFAR3 receptors on enteroendocrine L-cells and lowers GLP-1 secretion. Reduced GLP-1 availability deprives β-cells of key trophic and anti-apoptotic signaling mediated through the cAMP/PKA, EPAC2, and PI3K/AKT pathways (Tolhurst et al., 2012). Consequently, β-cell resilience to inflammatory and metabolic stressors declines, β-cell death accelerates, and the functional reserve of the islet compartment progressively deteriorates. Taken together, dysbiosis contributes to β-cell dysfunction through three mutually reinforcing mechanisms: LPS/TLR4-mediated inflammation, insulin-resistance–driven β-cell exhaustion, and reduced SCFA availability with consequent weakening of incretin signaling. Collectively, these axes establish an integrated pathophysiological framework linking microbiota disturbances to the progression of T2D and the progressive loss of β-cell function. Building on these mechanistic insights, multiple therapeutic strategies have been developed to counteract dysbiosis and restore a more favorable microbial environment.

## 7 The use of probiotics and other therapeutic approaches

Several strategies are available for correcting dysbiosis, including antibiotic therapy, dietary interventions, prebiotics, probiotics and their combined formulations, known as synbiotics. Antibiotic therapy is considered a short-term tool for selectively reducing the abundance of specific microbial groups, followed by remodeling of the intestinal ecosystem. Although such interventions can decrease inflammation and improve metabolic profiles, their clinical use is limited by the risk of dysbiosis and systemic side effects (Zarrinpar et al., 2018). A modern alternative to antibiotics includes phage therapy and anti-colonization strategies, which provide selective suppression of metabolically unfavorable taxa without significantly disrupting the overall microbiota structure. These approaches help reduce endotoxemia, strengthen the intestinal barrier, and decrease chronic inflammation, and are thus regarded as promising, highly precise microbiome-targeted tools for diabetes management. However, further clinical validation is still required (Ye et al., 2024).

Diet is a key determinant of gut microbiota composition (DiBaise et al., 2012). Dietary interventions, such as increasing fiber intake, restricting refined carbohydrates, and enriching the diet with fermentable substrates, promote a shift of the microbiota toward metabolically beneficial taxa without pharmacological intervention (Lee et al., 2019). Nevertheless, lifestyle modification poses considerable psychological challenges for many patients. Consequently, prebiotic- and probiotic-containing preparations are gaining popularity. Prebiotics are non-digestible food components that selectively stimulate the growth and metabolic activity of beneficial microorganisms such as Bifidobacterium and Faecalibacterium. This is accompanied by enhanced SCFA production, increased GLP-1 secretion, improved insulin sensitivity, better intestinal barrier integrity, and reduced systemic inflammation (Birkeland et al., 2020). Synbiotics, which combine probiotics and prebiotics, provide synergistic benefits through the simultaneous introduction of live microorganisms and substrates that support their selective growth. Several clinical trials have demonstrated their capacity to improve glycemic control, lipid profiles, and inflammatory markers in patients with T2D (Zhang et al., 2025).

The administration of probiotics such as *Lactobacillus plantarum* has been shown to restore microbiota balance, reduce inflammation, and improve β-cell function in murine models of diabetes (Zhong et al., 2024). Fecal microbiota transplantation (FMT) has also demonstrated efficacy in improving insulin sensitivity and restoring β-cell function in patients with T2D (Wu et al., 2023). Animal studies further indicate that gut microbiota directly regulate the regenerative potential of the epithelium, particularly by stimulating Lgr5^+^ stem cells and promoting differentiation of Paneth and goblet cells. For example, the probiotic strain *Limosilactobacillus reuteri* D8 activated the Wnt/β-catenin signaling pathway, increasing the number of Lgr5^+^ and lysozyme^+^ cells in the crypts, and stimulating epithelial proliferation both in the murine intestine and in organoid cultures (Wu et al., 2020). Similarly, 4 weeks of *Akkermansia muciniphila* administration accelerated Lgr5^+^ stem cell proliferation and enhanced Paneth and goblet cell differentiation in the small intestine, effects mediated *via* GPR41/43 receptor activation (Kim et al., 2021). Treatment with *Limosilactobacillus reuteri* ATCC PTA 4659 restored the expression of heat shock proteins Hsp70 and Hsp25 in the murine intestinal epithelium, improved tight junction structure, and reduced inflammatory responses, thereby strengthening the mucosal barrier and lowering systemic inflammation (Liu H.-Y. et al., 2022). Importantly, Lee Y.-S. et al. reported that daily oral administration of 4 × 10^9^ Colony-forming unit (CFU) of the probiotic *Lactobacillus plantarum* HAC01 exerted strong antidiabetic activity in a high-fat diet (HFD) mouse model, comparable to metformin. This treatment reduced islet atrophy, restored insulin^+^ β-cell area, and increased the abundance of Akkermansiaceae (*Verrucomicrobia*), paralleling the effects of metformin (Lee et al., 2021).

The efficacy of probiotics is largely attributable to their ability to modulate the “gut-liver-pancreas” axis. Restoration of the microbiota strengthens the intestinal barrier and reduces inflammation, which translates into systemic benefits and, consequently, improved β-cell function. Microbiota-derived butyrate activates AMPK/Akt signaling and suppresses gluconeogenesis. In the study by Liu Y. et al., oral administration of Lactiplantibacillus plantarum Y15 to STZ + HFD-induced T2D mice improved fasting glucose, HbA1c, and HOMA-IR levels, reduced pro-inflammatory cytokines (IL-6, TNF-α, among others), decreased the abundance of LPS-producing bacteria, and promoted SCFA-producing populations. This was accompanied by reduced inflammatory cell responses and enhanced insulin sensitivity through regulation of NF-κB-related genes and stimulation of the PI3K/Akt pathway (Liu Y. et al., 2022). A pronounced enhancement of PI3K/AKT signaling and increased SCFA production was also observed after 12 weeks of oral administration of a Lactobacillaceae strain mixture, isolated from healthy human feces, to db/db mice. The study by Li et al. demonstrated that this carefully selected bacterial mixture exerted robust, multifactorial antidiabetic effects in a T2D model. Probiotics reduced hyperglycemia, improved the morpho-functional state of the liver, kidneys, and pancreas, enhanced intestinal barrier function, and modulated inflammatory responses. By week 12, fasting blood glucose (FBG) levels were reduced by 22.4% compared to untreated control db/db mice. Histological analysis revealed significant improvements in the structure of the liver, kidneys, pancreas, and intestine. In the colon, reduced IL-1β mRNA levels were accompanied by increased IL-10 and ZO-1 expression, supporting barrier function. SCFA levels also rose markedly, particularly acetate and butyrate, whereas propionate levels showed only minor changes compared to controls (Li Y. et al., 2023).

Of particular interest are probiotic strategies involving strains engineered to express incretins, particularly GLP-1. The following section will discuss the design of such microorganisms, their mechanisms of action, and the outcomes of preclinical studies.

## 8 Gut microbiota and GLP-1-based therapeutics in diabetes

As noted earlier, modern bioinformatic approaches enable the integration of transcriptomic, proteomic, epigenomic, and microbiome data into a unified analytical framework, providing a multidimensional view of the molecular architecture of β-cells and their interactions with the microenvironment. The integration of multi-omics profiles with clinical parameters opens new avenues for the development of personalized therapeutic strategies in diabetes. In particular, the categorization of patients based on molecular subtypes of β-cell dysfunction not only refines our understanding of pathogenic mechanisms but also helps predict the efficacy of targeted interventions, including microbiome-modulating approaches and therapy with GLP1R agonists.

GLP-1 is one of the key hormones secreted by intestinal endocrine cells, primarily L-cells of the small intestine. It plays a central role in maintaining glycemic homeostasis and coordinating metabolic processes. GLP-1 secretion is glucose-dependent, and beyond its insulinotropic action, it suppresses glucagon release, delays gastric emptying, and reduces appetite, making it an important regulator of energy balance. Notably, GLP-1 also exerts trophic effects on β-cells, stimulating their proliferation, reducing apoptosis, and preserving their functional activity (Liu Y. et al., 2022). Interestingly, even in conditions of reduced GLP-1 secretion observed in individuals with obesity and T2D, its receptor-mediated effects remain intact. This observation provided the rationale for the development of pharmacological GLP1R agonists, which are now widely used in the treatment of T2D (Færch et al., 2015). The use of native GLP-1 as a therapeutic agent in T2D is hindered by its rapid inactivation in the body. Within 1–2 min after secretion, it is degraded by the enzyme dipeptidyl peptidase-4 (DPP-4), which sharply limits its therapeutic potential. This challenge spurred the development of not only degradation-resistant GLP1R agonists but also DPP-4 inhibitors, which prolong the half-life of endogenous GLP-1 by blocking the enzyme (Ling et al., 2019).

Gut microbiota can modulate GLP-1 secretion by influencing L-cell activity through metabolic products, primarily SCFAs, which activate FFAR2/3 receptors. This introduces an additional regulatory layer whereby the composition and activity of the microbiota directly affect intestinal hormonal activity and β-cell function (Tolhurst et al., 2012). Clinical evidence shows that GLP1R agonists not only improve glycemic control but also exert beneficial effects on body weight and the cardiovascular system, as confirmed in large-scale trials of agents such as Liraglutide (Marso et al., 2016), Dulaglutide (Gerstein et al., 2019), and Semaglutide (Lincoff et al., 2023). Thus, GLP-1-based therapy is evolving along both pharmacological and microbiome-related lines. On the one hand, the development of stable GLP1R agonists and DPP-4 inhibitors has overcome the limitations imposed by the short half-life of endogenous GLP-1, providing sustained action and clinically validated efficacy in improving glycemic control and reducing cardiovascular risk. On the other hand, growing evidence indicates that the gut microbiota acts as an additional regulator of GLP-1-dependent effects through metabolites such as SCFAs, which modulate L-cell activity and, indirectly, β-cell function. This opens the prospect of integrative approaches combining pharmacotherapy with microbiome modulation and establishes a foundation for novel strategies, such as genetically engineered probiotics capable of locally producing GLP-1 in the intestine. An overview of clinical and preclinical studies investigating microbiota-based interventions in T1D and T2D is summarized in Table 2 and 3, which highlights the diversity of approaches, ranging from probiotic supplementation and FMT to engineered bacterial strains.

**TABLE 2:** Preclinical and early-phase clinical studies of microbiota-based interventions in T1D and T2D.

Object of study	Study design	Number of participants	Main findings	References
Intake of high-amylose maize-resistant starch modified with acetate and butyrate (HAMSAB)	Pilot single-arm study, up to 12 weeks	25 adults with long-standing T1D screened; 20 initiated, 18 completed full 40 g/day dose for 6 weeks	Increased SCFAs in stool and plasma (acetate, propionate, butyrate), associated with improved HbA_1_c and reduced insulin dose; increase in *Bifidobacterium longum* and *B. adolescentis*, decreased pro-inflammatory cytokines, shift toward regulatory immune phenotype	Bell et al. (2022)
Associations between microbiota taxa and T1D risk (GWAS data)	Two-sample Mendelian randomization	18,340 individuals from the international microbiome consortium MiBioGen + summary statistic data for T1D (n = 264,137) from FinnGen consortium	Positive causal association of *Bacteroidetes/Bacteroidia/Bacteroidales* with T1D risk (OR≈1.24–1.28); *Eubacterium eligens* group protective (OR≈0.64); pleiotropy and heterogeneity nonsignificant	Luo et al. (2023)
Association between gut microbiota diversity and T2D/HOMA-IR	Population-based cohort (CARDIA sub-study), 16S sequencing, α- and β-diversity analysis	605 individuals: 417 non-diabetic, 101 prediabetic, 56 T2D+, 31 T2D−	Low α-diversity and altered β-diversity associated with higher HOMA-IR, longer disease duration and treatment; decreased butyrate producers (*Anaerostipes,* Lachnospiraceae*_UCG.004, Agathobacter, Faecalibacterium, Romboutsia*) in more severe T2D	Hu Y. H. et al. (2023)
FMT in T2D and diabetic kidney disease (DKD) mouse models	Preclinical experimental study; physiological, inflammatory, histological assessment, 16S rRNA analysis	6 animals per group: BTBR ob/ob, FMT+ and FMT−, BTBR WT control	FMT prevented weight gain, reduced albuminuria and TNF-α, improved insulin resistance and gut morphology; associated with abundance of succinate-consuming *Odoribacteraceae* across intestine	Bastos et al. (2022)
Capsule-based FMT in T1D with severe gastrointestinal symptoms	Randomized, double-blind, placebo-controlled pilot study; FMT capsules (∼25 capsules, 50 g feces) vs. placebo; crossover design	20 adult patients (10 FMT, 10 placebo)	FMT safe; significant improvements in GSRS-IBS, IBS-IS, and PAGI-SYM vs. placebo; more pronounced α- and β-diversity shifts in microbiota	Høyer et al. (2025)
Analysis of microbiota composition and T2DM subtypes	Mendelian randomization (two-sample MR analysis)	Large-scale GWAS microbiota/genotype datasets, tens of thousands of participants	Associations found: 6 taxa with severe insulin-deficient diabetes, 4 with severe insulin-resistant diabetes (SIRD), 8 with mild obesity-related diabetes (MOD), 8 with mild age-related diabetes; validation confirmed 4 taxa with stable effects, including Class *Clostridia* and Order *Clostridiales* (OR = 0.57, reduced MOD risk), *Catus* sp. (OR = 1.80, increased MOD risk), *Holdemania* (OR = 2.51, increased SIRD risk). No significant heterogeneity or pleiotropy	Ruan et al. (2025)

**TABLE 3:** Clinical studies of microbiota-based interventions in T1D and T2D.

Object of study	Study design	Number of participants	Main findings	References
Probiotic yogurt containing *Lactobacillus acidophilus* La5 and *Bifidobacterium lactis* Bb12	Randomized, double-blind, controlled clinical trial; 6 weeks of probiotic yogurt consumption (300 g/day)	64 patients with T2D, aged 30–60 years	In the probiotic yogurt group, there was a significant reduction in fasting glucose (P < 0.01) and HbA_1_c (P < 0.05), increased activity of erythrocyte superoxide dismutase and glutathione peroxidase, improved overall antioxidant status (P < 0.05), and decreased malondialdehyde concentration compared to baseline; effects were significant relative to control	Ejtahed et al. (2012)
Dairy product fermented with *Lactobacillus casei* strain Shirota (LcS)	Randomized, double-blind, placebo-controlled clinical trial; 8-week intervention with 4-week follow-up	100 prediabetic participants (BMI ≥25), randomized: 50 LcS group, 50 placebo; 98 completed the study	In the LcS group, significant reductions were observed in 1-h post-load glucose (p = 0.036), glycated albumin (p = 0.002), and HbA_1_c (p = 0.006); reduction in glycated albumin was greater than placebo (p = 0.030). Improvements were more pronounced in participants with higher baseline glycemia (p = 0.036, 0.034). Lipid profile improved: TC, LDL-C, and non-HDL-C were lower vs. placebo (p = 0.023; p = 0.022; p = 0.008)	Naito et al. (2018)
Multispecies probiotics or inulin-based synbiotic	Randomized, double-blind, placebo-controlled clinical trial	120 adults with prediabetes	Significant reduction in prevalence of hyperglycemia (p = 0.01/0.005), hypertension (probiotic, p = 0.04); reduced prevalence of metabolic syndrome and lower low HDL-C (probiotic, p = 0.02)	Kassaian et al. (2019)
Pasteurized *Akkermansia muciniphila*	Randomized, double-blind, placebo-controlled clinical trial; 3 months	40 overweight/obese patients with insulin resistance	Reduction in insulin resistance, decreased inflammatory markers, and improved metabolic profile	Depommier et al. (2019)
Longitudinal monitoring of 21 bacterial species in newly diagnosed T1D patients and autoantibody-positive relatives	Multicenter cohort study; ND cohort (≤24 months follow-up) and UFM cohort (36–48 months); metagenomic sequencing with laboratory monitoring	98 newly diagnosed T1D (ND cohort) and 194 unaffected family members (UFM cohort)	ND group: sustained increase in 21 bacterial species; *Faecalibacterium prausnitzii* negatively correlated with HbA_1_c (p = 0.0019); low α-diversity associated with rapid C-peptide decline; in some UFM who later developed T1D, *Sutterella* sp. KLE1602 increased (p = 1.2 × 10^−4^)	Vatanen et al. (2024)
Microbiome-modulating agents (probiotics, prebiotics, postbiotics, synbiotics) in T1D	Systematic review and meta-analysis of RCTs; quality assessment (Cochrane risk-of-bias), random-effects model	10 RCTs, ∼630 participants	Reduction in HbA_1_c, insulin use, and increased C-peptide, with no significant effect on glucose, inflammatory markers, lipids, or α-diversity; stronger HbA_1_c effect in trials >3 months	Zhang Y. et al. (2024)

Taken together, the clinical and preclinical studies summarized in Table X indicate that microbiota-targeted interventions generally produce modest but clinically measurable improvements rather than disease remission. Probiotic and synbiotic trials in T2D and prediabetes report statistically significant reductions in fasting glucose, HbA_1_c and surrogate markers of oxidative stress, yet the absolute changes are limited and do not obviate the need for standard glucose-lowering therapy (Li G. et al., 2023; Setayesh et al., 2025; Tao et al., 2020). Most trials and preclinical studies do not demonstrate consistent or clinically meaningful body-weight loss, suggesting a relatively weak impact on appetite regulation and energy balance, and where follow-up has been performed, beneficial effects tend to attenuate after discontinuation of the intervention (Soltani et al., 2023). In several cases, durable metabolic benefits appear to require co-administration with specific dietary substrates or prebiotics, such as inulin-based synbiotics or chemically modified resistant starch, highlighting that both microbial composition and nutrient context are critical for colonization stability and functional output (Wang et al., 2025).

### 8.1 Next-generation probiotics and recombinant forms of GLP-1

The concept of genetically modified probiotics is based on the creation of bacterial strains with predefined therapeutic functions that can synthesize biologically active molecules, such as hormones, cytokines, or peptides, directly within the gut. This approach integrates advances in synthetic biology and microbiome technologies, offering a targeted, localized, and minimally invasive therapeutic paradigm. In the context of diabetes, particular interest has been directed toward probiotics engineered to secrete GLP-1, aiming to stimulate insulin secretion and improve glycemic control.

Historically, one of the earliest strategies for creating recombinant long-acting GLP1R agonists involved the search for GLP-1 mimetics. Exendin-4 was the first natural GLP-1 mimetic, notable for its high resistance to DPP-4 degradation (Eng et al., 1992). Its synthetic form, exenatide, became the basis for the first approved GLP-1 agonists (U.S. Food and Drug Administration FDA, 2005). The pharmacokinetic properties of GLP1R agonists, which typically require injection, remain a factor limiting treatment adherence, thus highlighting the relevance of developing oral and other non-invasive formulations. One technological solution involves the use of a GRAS strain, *Lactobacillus paracasei*, engineered to express exenatide. This probiotic vehicle successfully induced insulin secretion, activated PDX1 and INS expression, and promoted β-cell proliferation through PI3K signaling, demonstrating efficacy comparable to native GLP-1 (Zeng et al., 2016). Another approach involves modifying *Lactobacillus plantarum* to produce native GLP-1, which was shown to improve metabolic parameters, reduce inflammation, and restore β-cell function in HFD/STZ mouse models of T2DM (Hu et al., 2023).

Of particular note is the ability of GLP1R agonists to induce transdifferentiation of intestinal cells into insulin-secreting cells. Initially, the potential for such transdifferentiation by native GLP-1, *via* activation of the Notch signaling pathway through NGN3 and downstream endocrine differentiation genes, was demonstrated by Suzuki et al. (Suzuki et al., 2003). Duan et al. were the first to show that oral administration of *Lactobacillus* engineered to express full-length GLP-1 (1–37) could induce intestinal epithelial cells to transdifferentiate into insulin-producing cells, restoring up to 33% of normal insulin secretory activity. Reprogrammed cells expressed key transcription factors of β-cell identity (Pdx1, MafA, FoxA2), while the morphology and function of the rat intestinal epithelium remained intact (Duan et al., 2015). As mentioned earlier, a key limitation of incretin therapy in diabetes is the short half-life of native GLP-1, which necessitates frequent administration. Thus, biological delivery systems capable of prolonged and physiologically relevant release of GLP-1 or its analogs are of particular interest. Lin et al. were the first to test a pentameric GLP-1 analog (5×GLP-1), expressed in *Lactobacillus paracasei* in both secreted and anchored forms with trypsin-cleavage sites. Despite its potential *in vitro*, *in vivo* therapeutic efficacy was limited due to insufficient peptide expression (Lin et al., 2016). This approach was refined in the work of Xu et al., who developed a recombinant GLP-1 analog - 6×mGLP-1 comprising six repeats of a mutated peptide resistant to both DPP-IV and trypsin. Produced in *E. coli*, 6×mGLP-1 demonstrated biological activity *in vitro*, stimulating proliferation of the mouse insulinoma cell line MIN6 even in the presence of trypsin, and *in vivo*, lowering blood glucose in diabetic mice. The effect was dose-dependent, persisted for up to 16 h after injection, and was accompanied by gradual release of active monomers. Oral administration produced a slower but still significant effect, confirming the promise of this approach for chronic therapy (Xu et al., 2017). In a recent study, a genetically modified strain of *E. coli* Nissle 1917 engineered to produce native GLP-1 showed sustained hypoglycemic effects in both T1D and T2D models. It also improved lipid metabolism, reduced inflammation, and protected β-cells from diabetes-associated cellular and systemic stress (Luo et al., 2025). These findings underscore that bacterial platforms expressing GLP-1 with controlled release can serve as an effective basis for long-term and physiologically coordinated diabetes therapy, while also highlighting the gut environment as a therapeutic target. However, GLP-1-expressing bacteria represent only one example of the broader concept of microbiota-mediated regulation of metabolic health.

## 9 Biosafety challenges and biological risks of engineered probiotic microorganisms

Despite encouraging preclinical results, a substantial translational gap remains between experimental findings and the clinical deployment of engineered probiotics. To date, no GLP-1–producing bacterial strain has progressed to human clinical trials. Regulatory agencies such as the FDA and EMA classify live modified microorganisms as higher-risk biological products, necessitating extensive preclinical safety evaluation, including assessment of genetic stability, exclusion of horizontal gene transfer, immunogenicity testing, and environmental biosafety analyses (Rouanet et al., 2020; U.S. FDA, 2016).

One of the principal concerns surrounding engineered probiotics is the potential for horizontal gene transfer, including the dissemination of GLP-1 expression cassettes or their regulatory elements into wild-type microbial populations. Such events could generate unpredictable phenotypes and disrupt intestinal ecosystem dynamics. Consequently, synthetic biology increasingly focuses on the development of multilayered biocontainment strategies that integrate metabolic dependencies, programmable genetic circuits, and multiple orthogonal triggers. As emphasized by Varma and colleagues, traditional single-layer kill-switch mechanisms often exhibit instability and may lose functionality within days, whereas multilayered systems—particularly those combining CRISPR-inducible modules, toxin–antitoxin systems, and synthetic auxotrophies—demonstrate markedly greater robustness, maintaining containment across hundreds of generations (Varma et al., 2025). Experimental studies further indicate that effective control typically requires the combination of synthetic auxotrophy, one or more kill-switch circuits, and CRISPR-based containment elements. For example, a multilayer biocontainment architecture developed for *Saccharomyces cerevisiae* and S. boulardii achieved an escape frequency below 10^−9^ and remained stable for over 100 generations, underscoring the feasibility of such systems for future live biotherapeutic applications (Maneira et al., 2025). These approaches are critical not only for preventing environmental dissemination but also for minimizing the risk of gene transfer to other gastrointestinal microbes.

Although the theoretical risk of prolonged colonization and chronic GLP-1 secretion is well-founded, there is currently no practical evidence from long-term clinical or preclinical studies demonstrating stable persistence, sustained GLP-1 production, or associated clinical effects. Existing data instead indicate generally low colonization stability even for well-studied probiotic strains: for example, *Escherichia coli* Nissle 1917 does not persist long-term in the human gut following oral administration (Charbonneau et al., 2020). Moreover, even under comparable conditions, bacterial strains exhibit marked inter-individual variability in their ability to colonize the intestine. Strains that engraft robustly in mice frequently show unpredictable or short-lived colonization in humans, and in certain patient groups—particularly individuals with T2D—engraftment appears even less stable (Tremblay et al., 2023; Zmora et al., 2018). This variability underscores another major limitation: the difficulty of dose standardization. Unlike injectable GLP-1 receptor agonists, bacterial systems exhibit high inter-individual variability in peptide expression and secretion, influenced by diet, baseline microbiota composition, and immune status. There is also a potential risk of immune responses to synthetic peptides or genetically modified strains, necessitating dedicated immunological safety assessment (Mousa et al., 2023). These factors indicate that GLP-1–expressing probiotics remain a proof-of-concept technology with substantial risks and unresolved translational barriers. Rigorous safety evaluation, development of human-relevant colonization models, and controlled early-phase clinical studies will be essential before engineered probiotics can be considered viable therapeutic candidates.

In addition to the systemic differences between rodents and humans, several mechanistic constraints further reduce the likelihood that GLP-1–secreting engineered probiotics will succeed clinically. First, bacterially produced GLP-1 often exhibits incomplete length or atypical processing because microorganisms lack the prohormone-convertase machinery (PC1/3, PC2) characteristic of enteroendocrine L-cells (Seidah et al., 2013). Second, GLP-1 secretion by engineered bacteria does not recapitulate the physiologically pulsatile release pattern of native L-cells, which depends on nutrient-derived stimuli, Ca^2+^ signaling, and coordinated enteroendocrine regulation (Galsgaard et al., 2025). Third, GLP-1 released into the intestinal lumen undergoes rapid degradation by pancreatic proteases and DPP-4, markedly reducing the concentration of active GLP-1 (7–36) before it can reach GLP1R-expressing target cells (Deacon, 2019). Taken together, these biophysical and pharmacokinetic limitations help explain why engineered GLP-1–producing strains demonstrate substantial efficacy in mice yet are unlikely to achieve comparable effects under clinical conditions.

## 10 Conclusion

Current evidence indicates that the functional heterogeneity and adaptive potential of β-cells in T2D are shaped by both intrinsic molecular programs and extrinsic regulatory cues, among which signals originating from the gut microbiota are particularly prominent. Through the production of metabolites such as short-chain fatty acids, indole derivatives, and bile acid–related molecules, and *via* receptor pathways including FFAR2/3, FXR, and TGR5, the microbiota modulates incretin secretion, tissue insulin sensitivity, immune tone, and the integrity of the intestinal epithelial barrier. Therapeutic strategies that target this axis, using probiotics, prebiotics, synbiotics, dietary interventions, or fecal microbiota transplantation, and, more experimentally, genetically engineered strains capable of local hormone or peptide delivery in the gut, offer the possibility of indirectly supporting β-cell function by attenuating metabolic stress and inflammation and restoring a more favorable incretin milieu.

At the same time, accumulating data from cell-based interventions indicate that durable remission of T2D will likely require strategies that go beyond metabolic control to directly preserve or rebuild β-cell mass. Early clinical experiences with implanted insulin-producing cells suggest that restoration of functional β-cell pools is feasible, but broad implementation will depend on overcoming barriers related to immune protection, standardization of cell manufacturing, and long-term safety and maturation of grafted cells. Taken together, these observations point to a future therapeutic paradigm that is inherently combinatorial: mitigation of metabolic and inflammatory stressors, protection and regeneration of endogenous β-cells, and, where necessary, β-cell replacement, complemented by targeted modulation of the gut microbiota.

## Glossary

T2D: Type 2 diabetes
scRNA-seq: Single-cell RNA sequencing
ER: Endoplasmic reticulum
scATAC-seq: Single-cell sequencing assay for transposase-accessible chromatin
NGPs: Next-generation probiotics
GLP-1: Glucagon-like peptide-1
GLP1R: Glucagon-like peptide-1 receptor
GIPR: Glucose-dependent insulinotropic polypeptide receptor
mRNA: Messenger RNA
cCREs: Candidate cis-regulatory elements
snRNA-seq: Single nucleus RNA sequencing
MODY3: Maturity-onset diabetes of the young, type 3
STZ: Streptozotocin
FAK: Focal adhesion kinase
PNM factors: PDX1, NGN3 and MAFA
EECs: Enteroendocrine cells
T1D: Type 1 diabetes
**AAb** ^**+**^: Autoantibody-positive
NK: Natural killer
TFs: Transcription factors
DEGs: Differentially expressed genes
SNPs: Single nucleotide polymorphisms
HPAP: Human Pancreas Analysis Program
WGBS: Whole-genome bisulfite sequencing
UPR: Unfolded protein response
DARs: Differentially accessible chromatin regions
HiChIP: High-throughput chromatin conformation capture with immuno precipitation
SCFAs: Short-chain fatty acids
GPCRs: G-protein-coupled receptors
Treg: Regulatory T cells
LPS: Lipopolysaccharides
FMT: Fecal microbiota transplantation
CFU: Colony-forming unit
HFD: High-fat diet
FBG: Fasting blood glucose
DPP-4: Dipeptidyl peptidase-4
ESCs: Embryonic stem cells
iPSCs: Induced pluripotent stem cells
